# Integrated meta-analysis, network pharmacology and experimental validation to explore the mechanism of traditional Chinese medicine against neonatal pneumonia: focus on naringenin/MAPK1

**DOI:** 10.3389/fped.2026.1824407

**Published:** 2026-06-04

**Authors:** Bo Gao, Bin Zhang, Peng Zhang

**Affiliations:** Neonatology Department, Panzhihua Women & Enfants Health Care Hospital, Panzhihua, Sichuan, China

**Keywords:** MAPK1, meta-analysis, naringenin, neonatal pneumonia, network pharmacology, traditional Chinese medicine

## Abstract

**Background and purpose:**

This study aimed to systematically analyze the clinical efficacy of traditional Chinese medicine (TCM) in treating neonatal pneumonia (NP), identify core active components and investigate the underlying molecular mechanisms, thereby providing reliable support for the clinical application of TCM in NP.

**Methods:**

A meta-analysis of seven clinical studies was conducted to identify effective TCM formulas. Network pharmacology approaches were applied to screen active ingredients, predict common targets, construct protein–protein interaction networks, and perform functional enrichment and molecular docking analyses. *In vitro* experiments using an LPS-induced BEAS-2B cell model were further performed to verify the anti-inflammatory effects and mechanisms of the key component.

**Results:**

A meta-analysis confirmed significant clinical efficacy of the TCM bath composed of *Perilla frutescens (L.) Britt.*, *Radix Bupleuri*, *Mentha haplocalyx Briq*., and *Schizonepeta tenuifolia Briq*. Six hub genes including MAPK1 were screened, and enrichment analysis indicated involvement in inflammatory and immune pathways. Molecular docking revealed relatively strong binding affinity between MAPK1 and naringenin, the key active component. *In vitro* experiments demonstrated that both naringenin and MAPK1 knockdown significantly enhanced cell viability and suppressed the production of the inflammatory cytokines IL-6, IL-17 and TNF-α. Furthermore, a mild synergistic effect was observed following combined treatment with naringenin and MAPK1 knockdown, compared with either intervention alone.

**Conclusion:**

Naringenin exerts therapeutic effects on NP partly by targeting MAPK1 and regulating inflammatory signaling pathways. Our systematic study provides novel theoretical and experimental evidence for the clinical application of TCM in NP.

## 1 Introduction

Neonatal pneumonia (NP) is the most common and life-threatening lower respiratory infection in the neonatal period. Its global morbidity and mortality remain high, with the mortality rate of perinatal infectious pneumonia ranging from approximately 5% to 20%. The burden is especially severe in preterm infants and very low birth weight neonates (< 1500 g), as well as in populations with low socioeconomic status, and is particularly concentrated in developing regions such as sub-Saharan Africa and South Asia (1–4). Due to immature respiratory systems, narrow airways, and low immune function, neonates are highly susceptible to bacterial, viral, and mycoplasmal infections, which can trigger uncontrolled inflammation and insufficient tissue repair, contributing to NP development (5, 6). With insidious onset, rapid progression, and atypical manifestations, NP easily leads to severe complications without timely intervention (7–10). Currently, the treatment of NP in modern medicine is mainly based on comprehensive supportive care. Bacterial infection is a major pathogenic factor of neonatal pneumonia, with *Klebsiella pneumoniae*, group B streptococcus, and *Escherichia coli* being prominent pathogens (11, 12), so broad-spectrum antibiotics such as ceftriaxone and penicillin are commonly used as first-line therapies (13, 14). Although these therapies improve outcomes to some extent, long-term antibiotic use induces drug resistance, intestinal flora imbalance, and adverse reactions (15, 16), and Western medicine alone is less effective in severe cases. Thus, exploring safe, effective, multi-targeted adjuvant therapies is crucial for NP management.

Traditional Chinese medicine (TCM) has exhibited distinctive advantages in the management of neonatal disorders and has been increasingly employed as an adjuvant intervention for pneumonia (17–20). Notably, the TCM system is abundant in various bioactive natural products, and many TCM herbs have been verified to possess anti-inflammatory and antibacterial activities, which are closely related to the pathological process of NP. For example, baicalein, a flavonoid compound, exerts a protective effect against NP by regulating the FOXA2/TRIM27-dependent pathway (21); resveratrol, a natural polyphenolic compound, significantly alleviates *Staphylococcus aureus*-induced pneumonia in mice, and its potential mechanism may be related to the inhibition of inflammasomes (22); hexahydrocurcumin, a component derived from *Zingiberis rhizoma*, could target and inhibit the JAK1/STAT3 signaling pathway to attenuate lipopolysaccharide-induced acute pneumonia; ganoderiol (23), a triterpenoid compound, reduces lung tissue damage in a rat model of pneumonia by decreasing the release of inflammatory mediators (24). In the field of TCM-based pneumonia therapy, numerous studies have investigated the therapeutic potential and mechanisms of medicinal herbs or herbal formulas. For instance, Wei et al. (25) demonstrated that the classic TCM formula Maxing Shigan Decoction exerts protective effects against pediatric bacterial pneumonia by regulating the HDAC3/NF-κB pathway and restoring short-chain fatty acid levels. Wang et al. (26) performed a meta-analysis of multiple clinical studies indicated that Xuebijing Injection, as an adjuvant therapy to Western medicine, could improve the total effective rate of severe pneumonia and reduce complications. Sun et al. (27) combined systematic pharmacology with literature data mining to screen out the main TCMs and their effective active components for the treatment of Mycoplasma pneumoniae, and analyzed the interactions among active compounds, drug targets, and signaling pathways to reveal the therapeutic effects of TCM. While these studies provide valuable insights, they still have limitations: most focus on a single herb or formula, fail to identify specific active components and core molecular targets, or lack clear connections and experimental evidence between clinical efficacy and underlying mechanisms.

In recent years, emerging technologies such as bioinformatics and network pharmacology are powerful tools for identifying TCM active components and therapeutic targets (28, 29). However, current studies are largely focused on classic traditional Chinese medicine prescriptions, with conclusions mainly derived from database mining and computational analysis, lacking robust experimental validation and clinical evidence. Meanwhile, most available clinical trials are limited to single-center, small-sample randomized controlled trials with variable methodological quality (30, 31). So high-quality systematic reviews and rigorous evidence-based medical evidence for Chinese patent medicines in the treatment of pneumonia are still lacking, especially in NP, which limits the clinical translation and application of Chinese patent medicines. In contrast, meta-analysis systematically integrates results from multiple independent clinical studies and yields more reliable, generalizable conclusions through quantitative synthesis. It emphasizes high-quality multicenter, large-sample clinical studies with long-term follow-up, and via rigorous evidence grading and systematic evaluation, provides scientific, objective evidence-based support for efficacy assessment, clinical medication optimization, and precise therapeutic strategy development, effectively addressing gaps in existing research (32–34).

Consequently, this study aimed to quantitatively evaluate the clinical efficacy of Chinese patent medicines in treating NP and explore their core active components, while elaborating on the underlying molecular mechanisms, through systematic analysis combined with experimental validation, so as to provide reliable theoretical evidence for clinical practice. Specifically, this meta-analysis focused on integrating multiple domestic studies on TCM treatment of NP, with particular attention to four traditional Chinese medicinal herbs with well-documented traditional medicinal value: *Perilla frutescens (L.) Britt.* (Zisu), *Radix Bupleuri* (Chaihu), *Mentha haplocalyx Briq.* (Bohe), and *Schizonepeta tenuifolia Briq.* (Jingjie). Concurrently, network pharmacology was integrated to construct herb-active component-target regulatory networks and protein-protein interaction (PPI) networks, thereby identifying core targets and initially clarifying potential molecular mechanisms through enrichment analysis. Collectively, this study is expected to lay a foundation for the rational clinical application of Chinese patent medicines in NP and further promote the integration of TCM theory with modern medical technologies.

## 2 Materials and methods

### 2.1 Meta-analysis

#### 2.1.1 Literature search strategy

This study aimed to investigate the relationship between NP and TCM. A comprehensive and systematic literature search was performed across four authoritative biomedical databases, including PubMed (https://pubmed.ncbi.nlm.nih.gov/), Embase (https://www.embase.com/landing?status=grey), the Chinese Biomedical Literature Database (CBM) (https://www.sinomed.ac.cn/index.jsp), and China National Knowledge Infrastructure (CNKI) (https://www.cnki.net/). The search strategy was constructed using a combination of subject headings (MeSH terms where applicable) and free-text keywords, with the core search terms including “neonatal pneumonia”, “newborn pneumonia”, “traditional Chinese medicine”, “Chinese patent medicine”, and “randomized controlled trial”. No restrictions were initially imposed on publication language, and both English and Chinese literatures were retrieved to ensure the comprehensiveness of the search. The advanced search functions of each database were utilized to combine the above keywords with logical operators (AND/OR) for precise retrieval, so as to identify all relevant randomized controlled trials (RCTs) that met the predefined inclusion criteria.

#### 2.1.2 Inclusion and exclusion criteria

**Inclusion criteria**: (1) Studies were limited to RCTs. (2) Participants were clinically diagnosed with neonatal pneumonia. (3) The intervention group received Chinese herbal medicine or Chinese patent medicines combined with conventional treatment, the control group received conventional Western medicine treatment, supportive care, or placeb. (4) Studies reported at least one of the clinical outcomes related to the efficacy of neonatal pneumonia treatment.

**Exclusion criteria**: (1) Non-RCT study designs, including observational studies, case reports, case series, and cross-sectional studies. (2) Studies without interventions of Chinese herbal medicine or Chinese patent medicines. (3) Studies with insufficient or unclear outcome data that could not be extracted for meta-analysis. (4) Reviews, systematic reviews, meta-analyses, animal experiments, *in vitro* studies, conference abstracts, theses, and theoretical studies. (5) Studies with serious methodological flaws, unclear grouping, or inappropriate interventions. (6) Studies with incomplete data, duplicate publications, unextractable outcome indicators obvious, and errors in data or inconsistent information that could not be verified.

#### 2.1.3 Study selection and data extraction

Two reviewers independently screened all retrieved literatures by reading titles and abstracts, obtained full texts of potentially eligible studies for further evaluation, and resolved disagreements by discussion or consultation with a third reviewer, with the study selection process recorded in a PRISMA flow diagram; two investigators also independently extracted data from the included studies using a standardized data extraction form, including basic information (first author, publication year, sample size, age or gestational age of neonates), intervention details (type of TCM, dosage, course of treatment, and treatment in the control group), outcome measures, and key methodological characteristics of each study.

#### 2.1.4 Risk of bias assessment and outcome measures

The methodological quality of each included RCT was assessed using the Cochrane Risk of Bias tool (RoB 1), evaluating seven domains (random sequence generation, allocation concealment, blinding of participants and personnel, blinding of outcome assessment, incomplete outcome data, selective reporting, and other potential biases) with each item graded as “low risk”, “high risk”, or “unclear risk”. In addition, the primary outcomes included the total effective rate and APACHE II score, and the secondary outcomes included time to disappearance of clinical symptoms (fever, cough, rales, etc.), length of hospital stay, levels of inflammatory factors, mortality and incidence of complications, and adverse effects.

#### 2.1.5 Statistical analysis

Statistical analysis was performed using RevMan 5.4 software. For dichotomous variables (clinical efficacy), odds ratio (OR) with 95% confidence interval (CI) was calculated to evaluate the pooled effect size. Heterogeneity among the 7 included studies was assessed using the *I*^2^ statistic: a fixed-effects model was adopted when *I*^2^ ≤ 50% (consistent with the low heterogeneity observed, *I*^2^ = 0.0%), while a random-effects model was reserved for potential high heterogeneity (*I*^2^ > 50%). Additionally, sensitivity analysis, subgroup analysis, and funnel plots were conducted to explore potential sources of heterogeneity and publication bias, respectively.

### 2.2 Network pharmacology analysis

#### 2.2.1 Drug-disease target network

To elucidate the potential molecular mechanisms underlying the therapeutic effects of TCM herbal interventions in neonatal pneumonia, network pharmacology analysis was integrated with our meta-analysis, with a specific focus on four core TCM herbs: *Perilla frutescens (L.) Britt*. (Zisu), *Radix Bupleuri* (Chaihu), *Mentha haplocalyx Briq*. (Bohe), and *Schizonepeta tenuifolia Briq*. (Jingjie). First, the active components of the four herbs and their corresponding potential molecular targets were systematically retrieved and screened from the Traditional Chinese Medicine Systems Pharmacology Database and Analysis Platform (TCMSP, https://www.tcmsp.org/), with default screening criteria for oral bioavailability and drug-likeness applied to ensure the reliability of candidate components. Concurrently, disease-related targets associated with neonatal pneumonia were comprehensively acquired from The Human Gene Database (GeneCards, https://www.genecards.org/) and Online Mendelian Inheritance in Man (OMIM, https://www.omim.org/); core targets of neonatal pneumonia were identified by intersecting the two disease target datasets to eliminate redundant and low-relevance targets. Subsequently, the overlapping targets between the candidate TCM herb targets and neonatal pneumonia core targets were obtained via intersection analysis, and a Venn diagram was constructed using the online bioinformatics analysis tool (Bioinformatics, http://www.bioinformatics.com.cn/) to intuitively visualize the common target sets.

#### 2.2.2 Construction of PPI network

STRING (https://string-db.org/), a dedicated online database for exploring PPI networks, was employed to analyze the common targets of the four TCM herbs and neonatal pneumonia. The overlapping target genes were imported into STRING version 11.0, with Homo sapiens designated as the reference organism and the interaction confidence score set to the high-confidence threshold of 0.900 to ensure the reliability of PPI pairs. The obtained PPI network data were then exported and further visualized using Cytoscape 3.7 software; in the constructed network, node size and color were dynamically adjusted based on the node degree values, where higher degree values corresponded to larger node sizes and distinct color gradients for intuitive identification of core nodes.

#### 2.2.3 Core target interaction network analysis

The PPI network was imported into Cytoscape software for visualization and in-depth topological analysis. To screen for core target genes, the cytoNCA plugin was utilized to calculate six key topological parameters, namely betweenness centrality, closeness centrality, degree centrality, eigenvector centrality, local average connectivity, and network centrality. A three-step sequential screening strategy was implemented, with only genes whose values for each parameter exceeded the median retained in each round. This stringent screening process ultimately identified core genes with the highest centrality values, which were defined as the potential key therapeutic targets of the four TCM herbs for neonatal pneumonia. In addition, the MCODE plugin in Cytoscape was employed to mine densely connected functional subnetworks within the PPI network. The analysis parameters were set as follows: degree cutoff = 3, node score cutoff = 0.2, and K-core = 3. The most significant functional subnetwork was extracted with a network score of 7.733, which represents a highly clustered and functionally conserved module. This module is hypothesized to be closely associated with the pathogenesis of neonatal pneumonia and may mediate the therapeutic effects of the TCM herbal interventions against the disease.

### 2.3 GO and KEGG enrichment analysis

The core target genes identified from the PPI network were imported into Metascape for functional enrichment analysis, which included Gene Ontology (GO) annotation and Kyoto Encyclopedia of Genes and Genomes (KEGG) pathway enrichment analysis. For GO analysis, the enrichment results were categorized into three ontologies: biological processes (BP), cellular components (CC), and molecular functions (MF). Statistical significance was determined by the *p*-value, and the top 10 significantly enriched terms for each GO ontology were screened out for subsequent analysis; for KEGG pathway analysis, the top 20 significantly enriched pathways were selected based on *p*-value ranking. All enrichment results were visualized using the online bioinformatics analysis platform (http://www.bioinformatics.com.cn/), with intuitive visualization plots generated to present the key enriched GO terms and KEGG pathways.

### 2.4 Molecular docking analysis

The three-dimensional (3D) crystal structures of the core target proteins were retrieved from the RCSB Protein Data Bank (PDB, https://www.rcsb.org/), and the canonical 2D/3D chemical structures of the candidate active compounds were obtained from the PubChem database. Prior to docking, the core target proteins were designated as receptor molecules and the TCM active compounds as ligand molecules; standard preprocessing was conducted for both receptors and ligands to ensure the reliability of docking results. Molecular docking was then performed between the preprocessed receptor proteins and ligand compounds, and all docking results were saved with the corresponding binding affinity values recorded in detail. Concurrently, a systematic analysis of the binding interaction modes (including hydrogen bonding, hydrophobic interactions, and ionic bonds) between the ligands and receptors was carried out. Binding affinity values served as the core quantitative indicator for evaluating the binding strength of ligand-receptor complexes; these values are inherently negative, with larger absolute values indicating stronger binding affinity between ligands and receptors. An absolute binding affinity value ≥ 10 kcal/mol was set as the screening threshold, representing favorable stability and robust binding activity of the formed ligand-receptor complex. Finally, the optimal molecular docking results were imported into PyMOL software (https://www.pymol.org/) for three-dimensional visualization and detailed analysis, which allowed for the intuitive presentation of key binding characteristics including binding sites, interaction forces and spatial conformation of the complexes.

### 2.5 Cell culture and treatment

Human bronchial epithelial cells (BEAS-2B) obtained from the American Type Culture Collection (ATCC) were cultured in high-glucose Dulbecco's Modified Eagle Medium (DMEM; 11965118, Gibco) supplemented with 10% fetal bovine serum (FBS; A5670401, Gibco). Cells at the logarithmic growth phase were digested with 0.25% EDTA-trypsin solution (25200114, Gibco), resuspended in fresh complete medium, and seeded into culture dishes. To establish an *in vitro* inflammatory model simulating the pathological state of pneumonia, cells were stimulated with 0.5 μg/mL lipopolysaccharide (LPS; L2880, Sigma-Aldrich) dissolved in normal saline and incubated at 37 °C in a 5% CO₂ atmosphere for 24 h. After successful model establishment, the cells were treated with naringenin (W530098, Sigma-Aldrich) at final concentrations of 2 μM, 10 μM, and 50 μM for 24 h.

### 2.6 Establishment of knockdown cell line

To knock down MAPK1 expression in BEAS-2B cells, a specific short hairpin RNA (shRNA) targeting MAPK1 (sh-MAPK1, target sequence: 5'-TGGAATTGGATGACTTGCCTA-3’) and a non-targeting scramble shRNA (negative control, sh-NC) were synthesized. The shRNA sequences were inserted into the pEGFP vector linearized with EcoRI and BamHI restriction enzymes. The constructed vectors were transfected into competent E. coli cells, and positive clones were identified by PCR and sequencing to obtain the recombinant MAPK1-shRNA plasmid. BEAS-2B cells in the logarithmic growth phase were transfected with the recombinant MAPK1-shRNA plasmid, negative control plasmid, and empty pEGFP vector using Lipofectamine 3,000 reagent. After 48 h of transfection, the knockdown efficiency of MAPK1 at the mRNA and protein levels was verified by RT-qPCR and Western blotting analysis, respectively. Cells with efficient MAPK1 knockdown were used for subsequent experiments.

### 2.7 CCK-8 assay

Cell viability was measured using a CCK-8 assay kit (C0038, Beyotime, Shanghai) in 96-well plates (CLS3922, Corning). BEAS-2B cells at the logarithmic growth phase were harvested, resuspended in complete medium, and seeded at an appropriate density in a final volume of 100 μL. After incubation at 37 °C in a 5% CO₂ atmosphere for 24 h, the cells received corresponding model establishment and intervention treatments according to different groups. Then, 10 μL of CCK-8 solution was added to each well, followed by incubation for another 2 h. The absorbance at 450 nm was detected using a microplate reader (DNM-960, Prang, Beijing) to evaluate cell viability.

### 2.8 ELISA

The concentrations of tumor necrosis factor-α (TNF-α, SEKM-0034), interleukin-6 (IL-6, SEKM-0007), and interleukin-17 (IL-17, SEKH-0026) in cell culture supernatants were measured using commercial ELISA kits (Beijing Solarbio Technology Co., Ltd., China). All procedures were performed strictly according to the manufacturer's instructions. After incubation with samples and detection antibodies, 3,3’,5,5'-tetramethylbenzidine substrate was added for color development, and the reaction was stopped using a stop solution. The absorbance at 450 nm was measured using a microplate reader, and cytokine concentrations were calculated based on standard curves with four-parameter logistic fitting.

### 2.9 RT-qPCR

Total RNA was extracted from BEAS-2B cells using TRIzol reagent (Majorbio Technology Co., Ltd., Shanghai, China) according to the standard protocol. Then the concentration and purity of the isolated RNA were determined. cDNA was synthesized from equal amounts of total RNA using the FastKing RT kit (Majorbio Technology Co., Ltd., Shanghai, China) in a standard reverse transcription system. RT-qPCR was then performed using the SYBR Green Premix Pro Taq HS qPCR kit (Accurate Biotechnology Co., Ltd., Changsha, China) on a real-time fluorescence quantitative PCR instrument. The relative mRNA expression levels of target genes were calculated using the 2^(-ΔΔCt) method with *GAPDH* as the internal reference gene. All reactions were performed in triplicate to ensure the reliability and reproducibility of the results. The specific sequences of the primers are listed in Table 1.

**Table 1:** Primer sequences of the target gene.

Name (Homo sapiens)	Forward primer (5′→3′)	Reverse primer (5′→3′)
TNF-α	GCCTCTTCTCATTCCTGCTT	TGGGAACTTCTCATCCCTTTG
IL-6	CAGACCAGTATATACCACTTC	ATATCCAGTTTGGAAGCATCC
IL-17	AGATGAAGCTCTCCCTGGACTCAT	ATCCACCTCACACGAGGCACAA
GAPDH	TGATGCCCCCATGTTTGTGA	TTCTGAGTGGCAGTGATGGC

### 2.10 Western blotting

BEAS-2B cells were washed with pre-chilled PBS and lysed in RIPA buffer (G2002, Servicebio, China) containing protease inhibitors (G2008, Servicebio, China). The protein supernatant was collected by centrifugation at 12,000 × g for 10 min, and the protein concentration was determined using a BCA assay kit (20200ES76, YEASEN, China). Equal amounts of protein were separated by 12% SDS-PAGE and transferred to PVDF membranes (IPVH00010, Millipore,USA). Membranes were blocked with 5% skim milk at room temperature for 2 h, then incubated overnight at 4 °C with primary antibodies against TNF-α (1:500, AF7014), IL-17 (1:1000, DF6127), IL-6 (1:1000, DF6087), and GAPDH (1:3000, AF7021) (all from Jiangsu Qinke Biological Research Center, China). After three washes, membranes were incubated with HRP-conjugated sheep anti-rabbit IgG secondary antibody (1:8000, Abcam, UK) for 1.5 h at room temperature. Protein bands were visualized using Western Lightning™ chemiluminescence reagent (G2086, Servicebio, China) and quantified by optical density analysis, with GAPDH as the internal reference.

### 2.11 Statistical analysis

Statistical analyses were performed using GraphPad Prism V8.0 software. All experimental data are presented as mean ± standard deviation, derived from at least three independent experiments. Comparisons between two groups were performed using paired t-tests for data meeting the assumptions of normality and homogeneity of variance. For comparisons among three or more groups, one-way analysis of variance (ANOVA) was applied, followed by Tukey's HSD *post hoc* test for multiple comparisons. A *p*-value < 0.05 was considered statistically significant (*p < 0.05, **p < 0.01, ***p < 0.001, ****p < 0.0001).

## 3 Results

### 3.1 Meta-analysis

**Figure 1:**
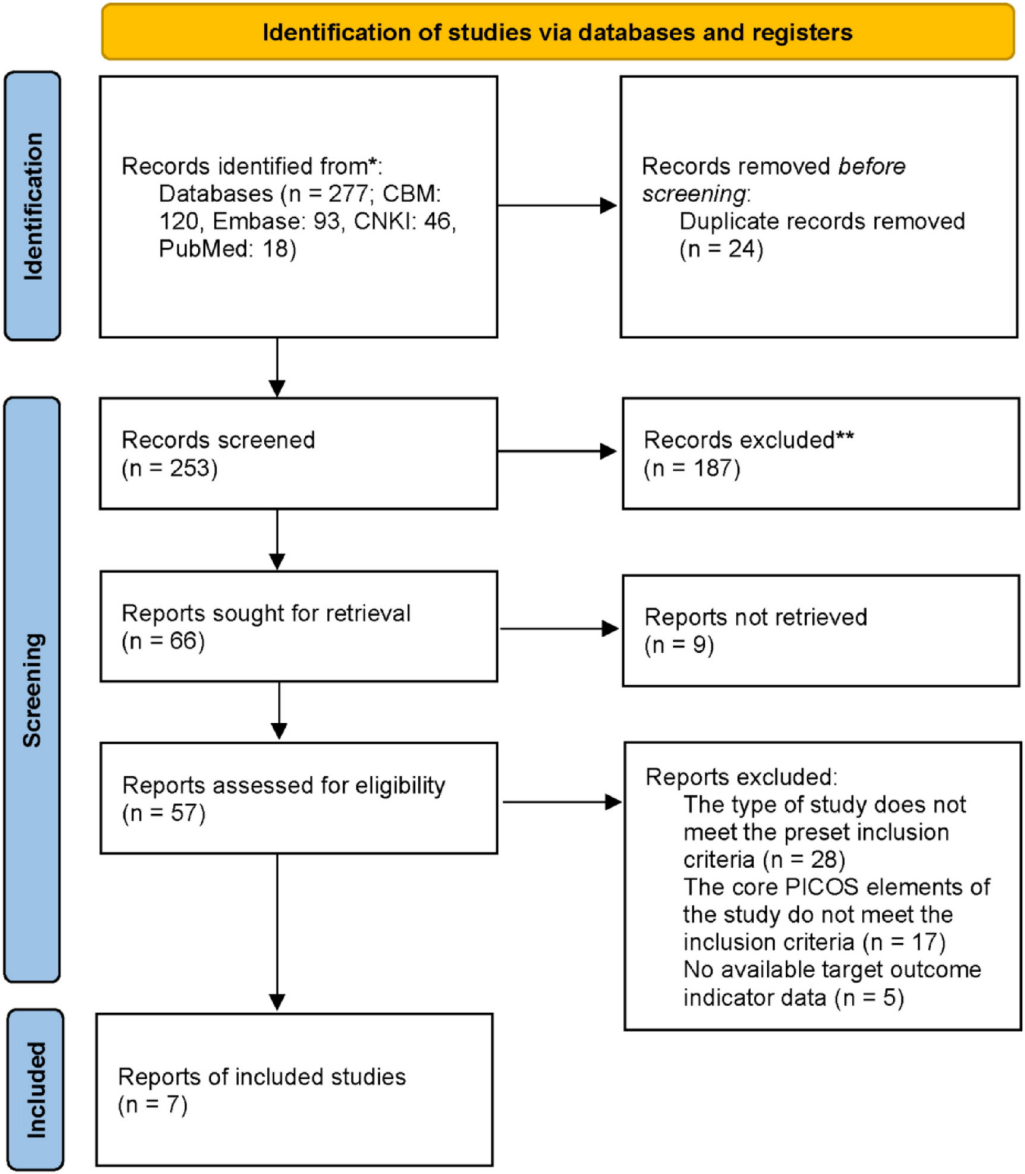
PRISMA flow diagram of literature screening and selection process. A total of 277 relevant studies were initially identified from four databases, including 120 from CBM, 93 from Embase, 46 from CNKI, and 18 from PubMed. After stepwise screening of duplicates, titles, abstracts and full texts, 7 Chinese studies that fully met the inclusion and exclusion criteria were finally included in the meta-analysis.

**Figure 2:**
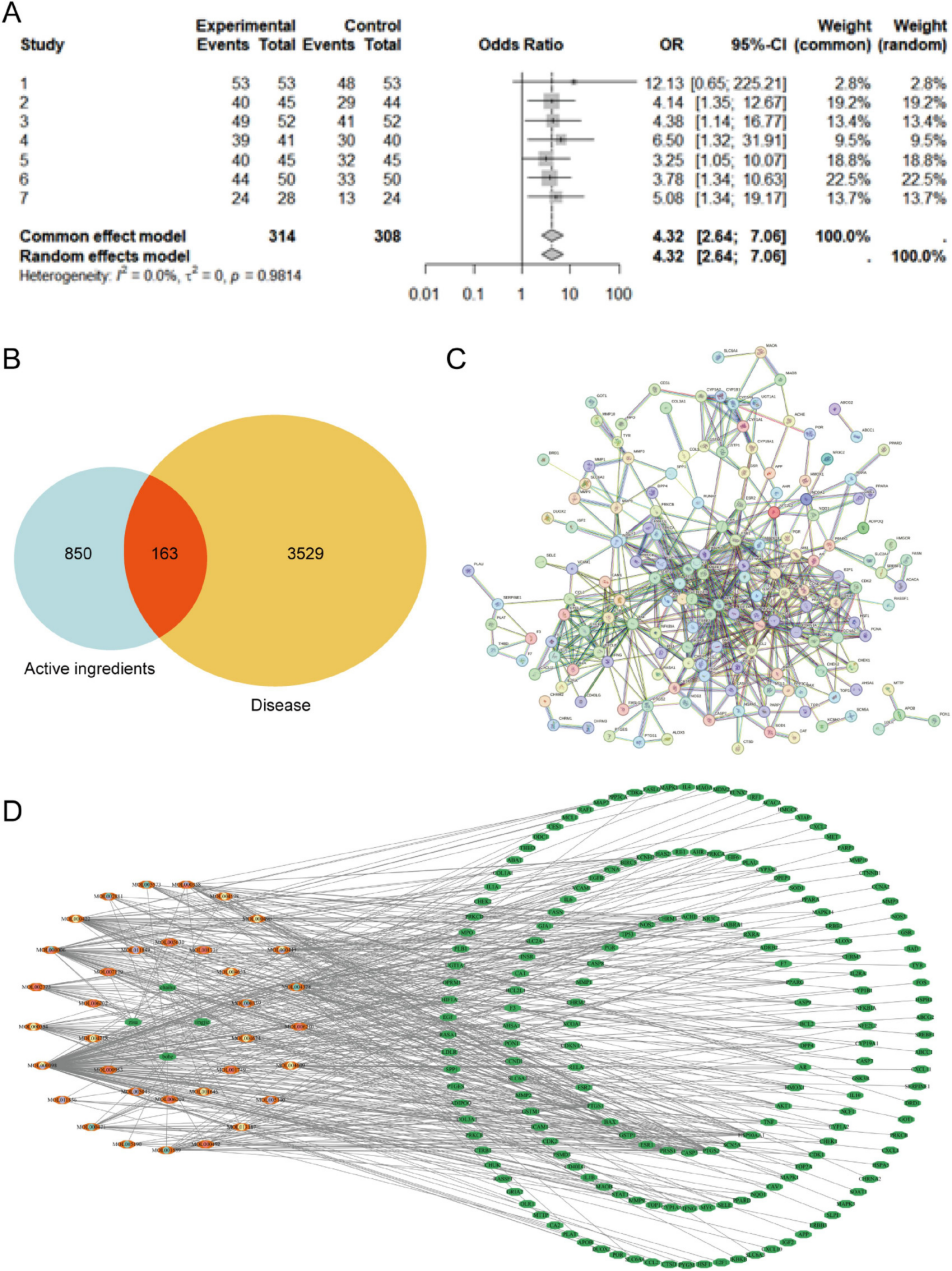
Meta-analysis and network pharmacology reveal common targets and regulatory interaction networks of herbal medicines for NP. **(A)** Forest plot of the meta-analysis evaluating the clinical efficacy of Chinese herbal medicine interventions for NP. **(B)** Venn diagram showing the intersection between herb-related targets and NP-related disease targets. **(C)** PPI network of the common targets. **(D)** The “Chinese herbal medicines–active ingredients–common targets” regulatory network. Green nodes on the right represent common targets; red nodes on the left represent active ingredients; and blue nodes on the left represent the four herbal medicines (Chaihu, Jingjie, Bohe, Zisu). Lines represent interactions between nodes.

A meta-analysis was conducted to assess the clinical efficacy of the TCM interventions. The PRISMA flow diagram (Figure 1) illustrated the literature screening process and final inclusion results, with a total of 7 eligible studies ultimately enrolled, involving 314 patients in the treatment group and 308 patients in the control group (Table 2). Statistical analyses were performed using both fixed-effects and random-effects models to ensure the stability and reliability of pooled estimates. The pooled OR was calculated to be 4.32, with a corresponding 95% CI of [2.64, 7.06], indicating that theses interventions were associated with a significantly improved clinical response compared with the control regimen. Moreover, heterogeneity assessment revealed low between-study variability (*I*^2^ = 0.0%, *p* = 0.9814), indicating high consistency and reliability across the included studies. Notably, the study titled “Efficacy observation of traditional Chinese medicine bath in the treatment of fever in neonatal pneumonia” exhibited the highest OR value, implying the strongest association between the intervention and improved clinical outcomes in neonatal pneumonia. Based on the frequency and importance of herb composition in the highly effective studies, four core herbal components were identified as key candidates for subsequent network pharmacology analysis: Zisuye, Chaihu, Bohe, and Jingjie (Figure 2A).

**Table 2:** Principal characteristics of all included studies in meta-analysis.

NO.	Author year	Literature	Medicinal materials	Treatment methods
1	2021 Zhou Fang	Observation on the Therapeutic Effect of Traditional Chinese Medicine Bath in Neonatal Pneumonia with Fever (35)	*Perilla frutescens (L.) Britt., Radix Bupleuri, Mentha haplocalyx Briq., and Schizonepeta tenuifolia Briq.*	TCM Herbal Bath
2	2020 Yin Xiangqin	The Effect of Applying Fubei Powder to Shuangfeishu to Assist Western Medicine in the Treatment of Neonatal Pneumonia (36)	*Rheum palmatum L., Natrii Sulfas, Rumex acetosa L.*	Fubei Powder was applied to both Feishu acupoints
3	2019 Liu Wei	Clinical Observation on Traditional Chinese Medicine Acupoint Application on Neonatal Pneumonia (37)	*Salvia miltiorrhiza Bge., Borneolum Syntheticum, Corydalis yanhusuo W. T. Wang, Sinapis Albae*	Acupoint Application of TCM
4	2017 Ou Caixiang	Observation on efficacy of acupoint application of Chinese medicine in the treatment of neonatal pneumonia (38)	*Ephedra sinica Stapf, Asarum sieboldii Miq., Sinapis Albae, Corydalis yanhusuo W. T. Wang, Zingiberis Rhizoma*	Acupoint Application of TCM
5	2018 Leng Dongming	Observation on the Therapeutic Effect of Acupoint Application of Traditional Chinese Medicine as an Adjuvant Treatment for Neonatal Pneumonia (39)	*Salvia miltiorrhiza Bunge, Borneolum Syntheticum, Corydalis yanhusuo W.T.Wang, Sinapis Albae, Mel*	Acupoint Application of TCM
6	2013 Jiang Li	Clinical efficacy of Zhen Wu Tang combined with western medicine in treatment of neonatal pneumonia with pulmonary hypertension (40)	*Aconiti Radix Lateralis Praeparata, Poria Cocos, Zingiberis Rhizoma, Paeoniae Radix Alba, Atractylodis Macrocephalae Rhizoma, Glycyrrhizae Radix Et Rhizoma Praeparata Cum Melle*	Oral administration
7	2007 Chang Xiaojun	Observation of the Therapeutic Effect of Transdermal Drug Administration in Neonatal Pneumonia (41)	*Houttuyniae Herba, Lonicerae Japonicae Flos, Isatidis Radix, Armeniacae Semen, Platycodonis Radix, Fritillariae Cirrhosae Bulbus*	Topical Application of TCM

### 3.2 Network pharmacology analysis

The four identified herbal components were subsequently submitted to the TCMSP for the screening of pharmacologically active ingredients and their corresponding putative drug targets. During the screening process, ingredients with favorable pharmacokinetic properties were retained, yielding a total of 36 active components (including naringenin, acacetin, and petunidin, among others) and 1,013 unique drug targets associated with these herbs. Meanwhile, disease-related targets linked to neonatal pneumonia were collected from GeneCards and OMIM databases. After removing duplicate entries and conducting overlap screening, a final set of 3,692 high-confidence disease targets was obtained. To identify key targets responsible for the therapeutic effects, intersection analysis was performed between herb-derived drug targets and neonatal pneumonia-related targets, identifying 163 shared common targets (Figure 2B). These 163 common targets were further uploaded to the STRING database for PPI analysis. To ensure high reliability and biological relevance, only interactions with a confidence score > 0.9 were included in the final PPI network (Figure 2C). Finally, Cytoscape software was employed to construct and visualize a comprehensive “Chinese herbal medicines–active ingredients–common targets” regulatory network, which intuitively illustrated the multi-component, multi-target mechanism of the herbal intervention against neonatal pneumonia (Figure 2D).

### 3.3 GO and KEGG enrichment analysis

**Figure 3:**
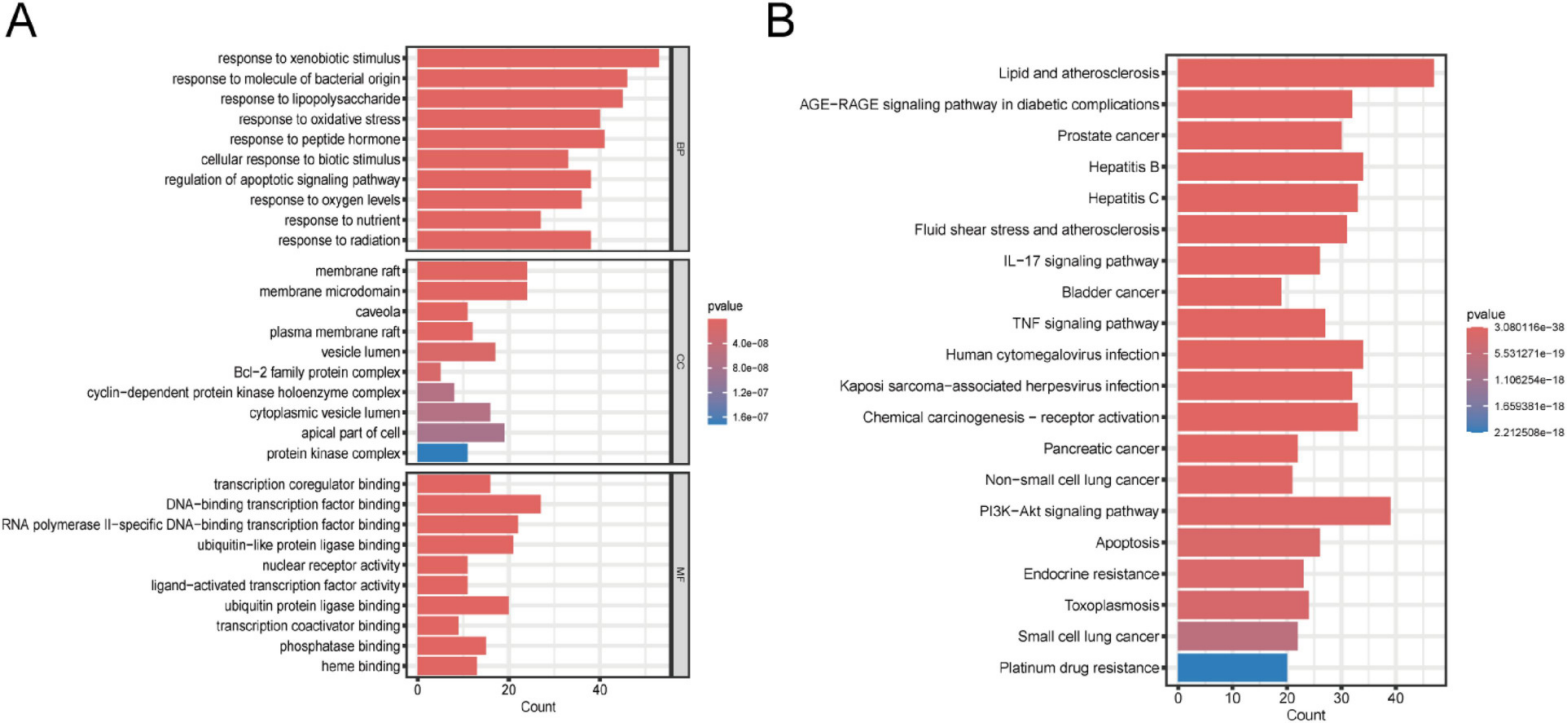
Functional enrichment analysis of common targets. **(A)** GO enrichment bar plot. **(B)** KEGG pathway enrichment bar plot.

To further clarify the potential molecular mechanisms by which the four herbal components exert therapeutic effects on NP through their key common targets, GO enrichment analysis and KEGG pathway enrichment analysis were performed on the 163 identified common targets. In the BP category, the common targets were significantly enriched in biological processes closely related to the pathological progression of NP, including response to xenobiotic stimulus, response to molecules of bacterial origin, and response to lipopolysaccharide—these processes are closely associated with the inflammatory response and immune regulation. For the CC category, the most prominently enriched cellular components were membrane rafts, with additional significant enrichment in membrane microdomains and the apical part of the cell; these components are involved in cell signal transduction and the regulation of inflammatory factor secretion, providing a structural basis for the interaction between active ingredients and key targets. In terms of MF, the key common targets were mainly enriched in DNA-binding transcription factor activity, RNA polymerase II-specific DNA binding, transcription factor binding, and ubiquitin-like protein ligase binding—these molecular functions are critical for regulating gene transcription, cell proliferation and apoptosis, and inflammatory response, further supporting the multi-target regulatory mechanism of the herbal components in treating NP (Figure 3A). KEGG pathway enrichment analysis revealed that among the top 20 enriched pathways, the most significant enrichment was observed in pathways such as lipid and atherosclerosis, PI3K-Akt signaling pathway, TNF signaling pathway, and IL-17 signaling pathway—these pathways are well-documented to be involved in the regulation of inflammatory responses, immune cell activation, and pulmonary tissue damage, which are key pathological processes in neonatal pneumonia. Additionally, pathways including the RAGE signaling pathway in diabetic complications, prostate cancer, and lipid and atherosclerosis had the largest number of enriched common targets, indicating that the herbal components may exert their therapeutic effects on NP by regulating these multi-pathway synergistic networks (Figure 3B).

### 3.4 Core targets interaction network analysis

**Figure 4:**
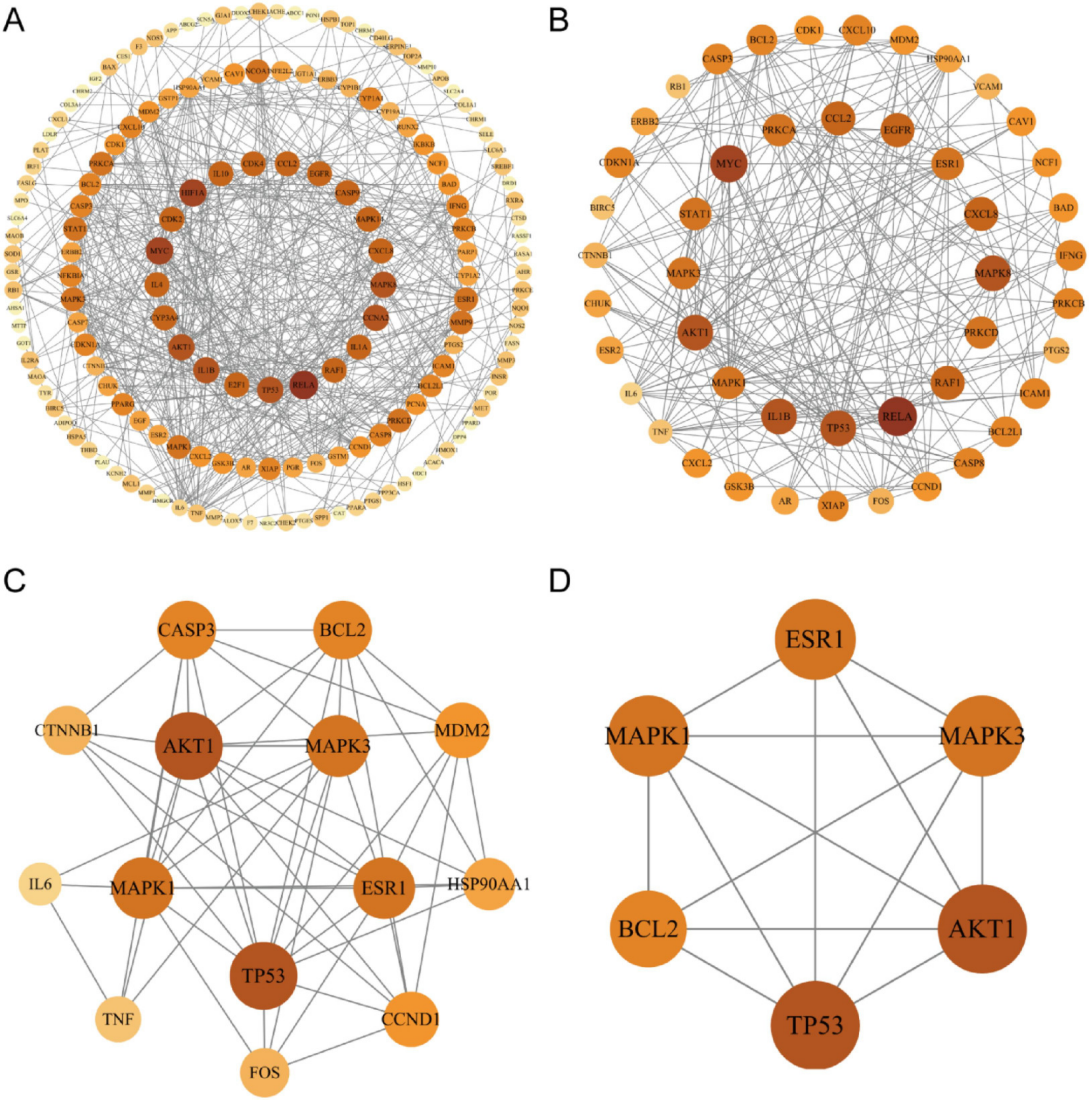
Construction of the PPI network and identification of hub genes by topological analysis. **(A)** Initial PPI network structure. **(B–D)** Final six core genes identified through three rounds of filtering based on six topological properties.

To further identify the core regulatory targets that mediate the effects of the active ingredients against NP, topological analysis of the PPI network was performed using the cytoNCA plugin in Cytoscape software. A total of six topological parameters were applied for hub gene screening, including Betweenness, Closeness, Degree, Eigenvector, Local Average Connectivity (LAC), and Network Centrality. For each parameter, only targets with values greater than the median threshold were regarded as candidate targets and retained for further analysis (Table 3). Following a three-step progressive screening process, six targets with the highest topological importance and biological relevance were ultimately identified as hub genes. These key targets were MAPK1, ESR1, TP53, AKT1, BCL2, and MAPK3, which were considered to play central roles in the therapeutic mechanism of the four herbal medicines against NP (Figures 4A–D).

**Table 3:** Parameter of core genes.

Gene	Betweenness	Closeness	Degree	Eigenvector	LAC	Network
ESR1	7.871	0.765	9	0.310	4.889	7.051
TP53	10.957	0.867	11	0.373	6.182	10.382
MAPK1	13.618	0.765	9	0.304	4.667	6.461
AKT1	16.759	0.867	11	0.366	5.818	9.775
MAPK3	14.831	0.765	9	0.291	4.444	6.911
BCL2	3.805	0.722	8	0.296	5.000	6.133

### 3.5 Identification of key subnetworks and primary target

**Figure 5:**
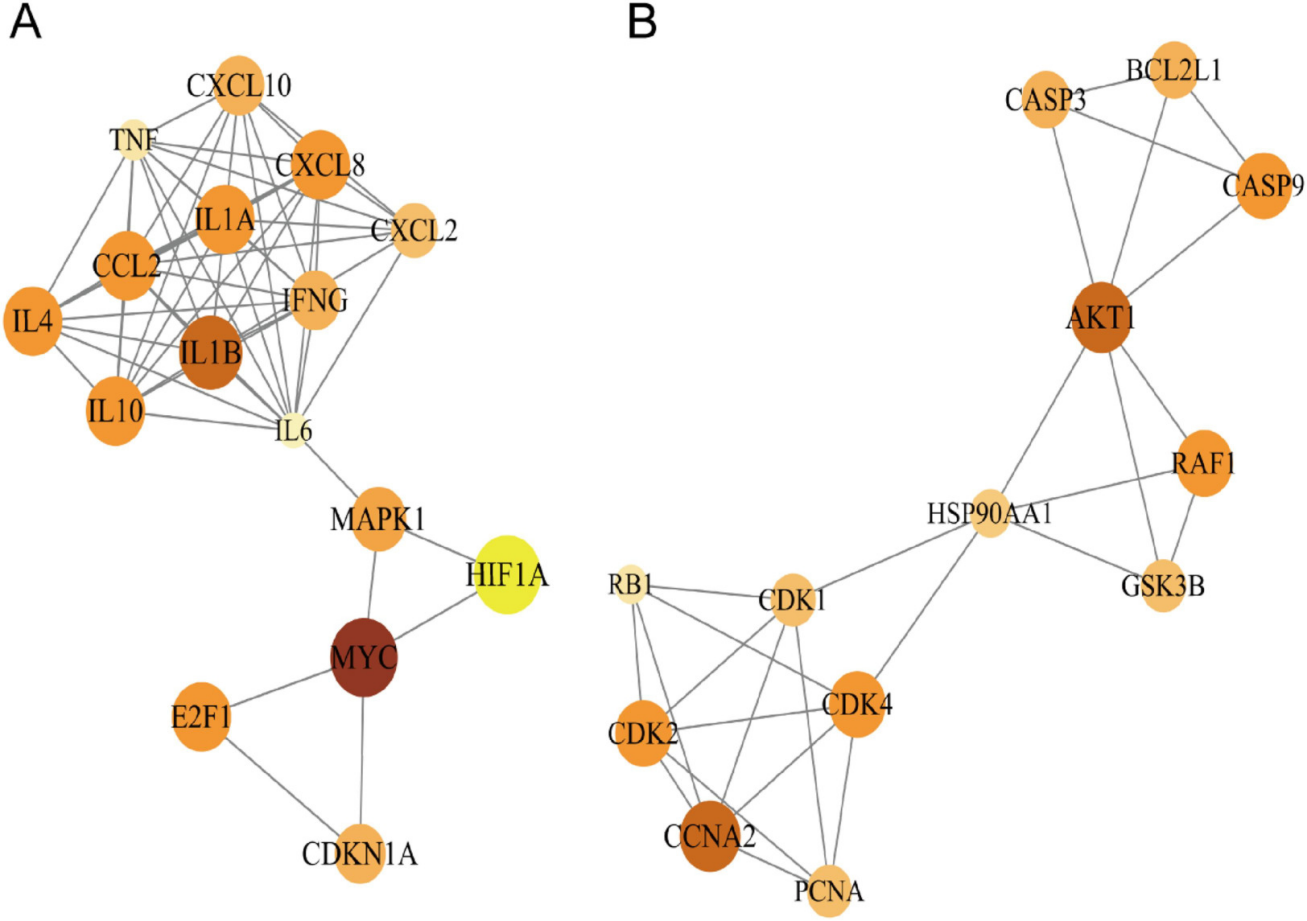
MCODE plugin identification of core subnetworks. **(A)** Subnetwork A identified through PPI network analysis. **(B)** Subnetwork B identified through PPI network analysis.

The MCODE plugin embedded in Cytoscape software was employed to conduct module analysis of the 163 common targets, aiming to identify core functional subnetworks that play critical roles in the therapeutic mechanism of the four herbal components against NP. Module analysis results identified two key functional subnetworks with distinct MCODE scores and target compositions. Subnetwork A (MCODE score = 7.733) consisted of MAPK1, CXCL10, TNF, CXCL8, and IL4 (Figure 5A), which are mainly involved in inflammatory and immune responses. Subnetwork B (MCODE score = 4.5) included AKT1, CASP3, BCL2L1, and RB1 (Figure 5B), associated with apoptosis and cell proliferation. By comprehensively considering the MCODE scores, topological core target characteristics, and KEGG enrichment results highlighting the TNF and IL-17 signaling pathways, MAPK1 was chosen as the primary hub target for subsequent validation.

### 3.6 Molecular docking analysis

**Figure 6:**
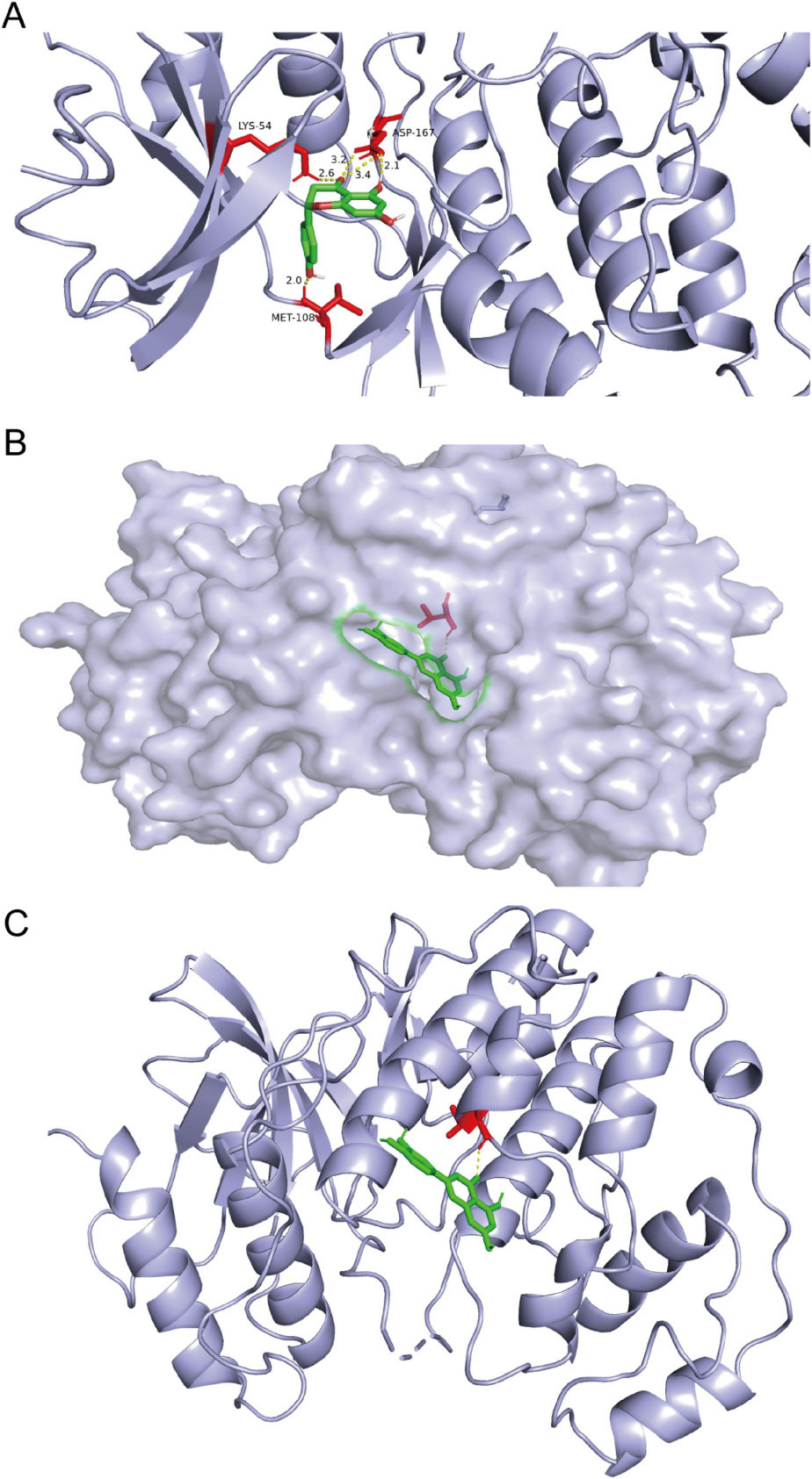
Molecular docking structural visualization. **(A–C)** 3D representation of the spatial interaction between the protein and the active compound naringenin (color-coded) from different angles.

To explore the potential binding mechanism between MAPK1 and the key active components derived from the four herbal medicines, molecular docking was performed between MAPK1 protein and representative compounds. The binding affinity, docking conformation, and intermolecular interactions were comprehensively evaluated. The docking results demonstrated that naringenin exhibited the strongest binding stability with MAPK1, with a calculated binding energy of −7.5 kcal/mol. These results indicated a stable and tight interaction between naringenin and MAPK1, which provides a structural basis for further investigation into the pharmacological mechanism by which naringenin targets MAPK1 against neonatal pneumonia (Figures 6A–C).

### 3.7 Efficacy of naringenin in a BEAS-2B neonatal pneumonia model

**Figure 7:**
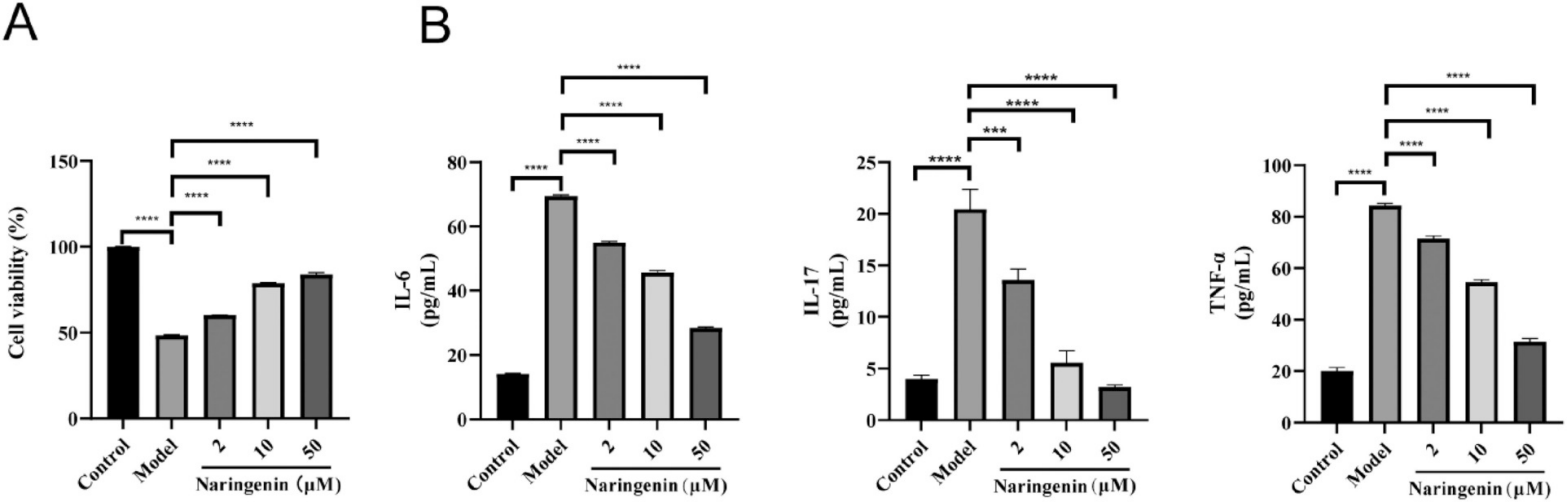
Effects of naringenin on cell viability and inflammatory cytokine secretion in LPS-stimulated BEAS-2B cells. **(A)** Cell viability was detected by CCK-8 assay after treatment with different concentrations of naringenin (2 μM, 10 μM, 50 μM). **(B)** The levels of pro-inflammatory cytokines (IL-6, IL-17, TNF-α) were measured by ELISA. Data are presented as mean ± SD (*n* = 3); ****P < 0.0001 vs. the control and model group.

Based on the aforementioned network pharmacology and molecular docking analyses, we first established an *in vitro* BEAS-2B pneumonia cell model to verify the regulatory effects of naringenin on neonatal pneumonia. Cell viability was detected using the CCK-8 assay, and the results showed that compared with the normal control group, the BEAS-2B cells in the neonatal pneumonia model group exhibited a significant reduction in cell viability (*p* < 0.0001). In contrast, treatment with naringenin at gradient concentrations significantly reversed the decrease in cell viability, and this restorative effect presented a clear concentration-dependent pattern (*p* < 0.0001) (Figure 7A). In addition, ELISA was performed to detect the secretion levels of pro-inflammatory cytokines, and the results demonstrated that the expression levels of IL-6, IL-17, and TNF-α in the model group were significantly higher than those in the control group (*p* < 0.0001). Notably, naringenin treatment significantly inhibited the excessive release of these three pro-inflammatory cytokines, and the inhibitory effect was also concentration-dependent (*p* < 0.0001) (Figure 7B). Collectively, these *in vitro* experimental results clearly demonstrate that naringenin enhances cell viability and suppresses the inflammatory response in neonatal pneumonia-related BEAS-2B cells in a concentration-dependent manner, which further validates the potential therapeutic effect of naringenin on neonatal pneumonia.

### 3.8 Functional validation of MAPK1 in neonatal pneumonia cell model

**Figure 8:**
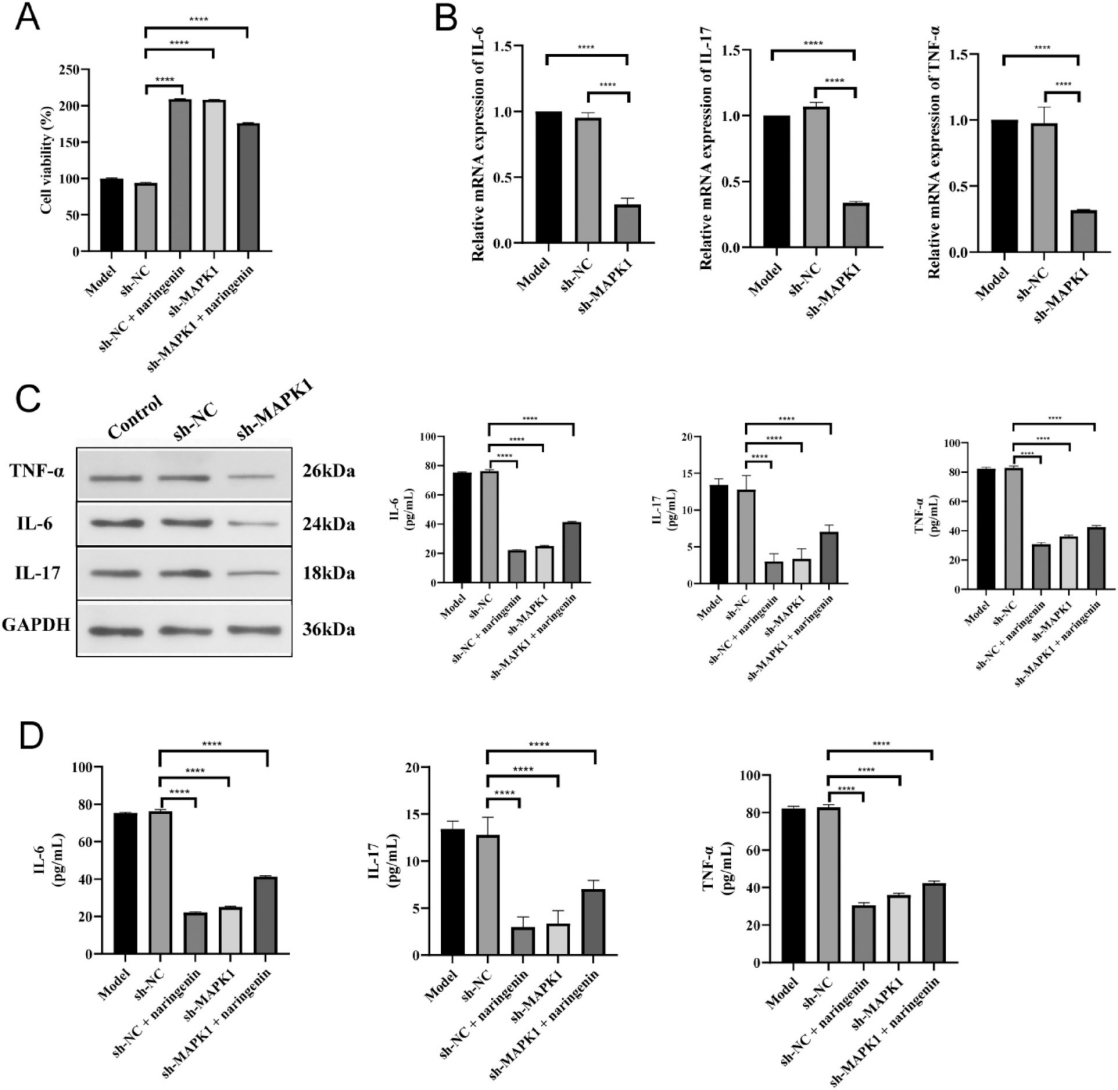
Effects of MAPK1 knockdown on inflammatory responses and cell viability in LPS-stimulated BEAS-2B cells. **(A)** Cell viability assessed by CCK-8 assay. **(B)** Relative mRNA expression levels of pro-inflammatory cytokines detected by RT-qPCR. **(C)** Protein expression levels of pro-inflammatory cytokines detected by western blotting. **(D)** The secretion levels of IL-6, IL-17, and TNF-α in each group were measured by ELISA. Data are presented as mean ± SD (*n* = 3); ****P < 0.0001 vs. the model or sh-NC group.

To investigate the role of MAPK1 in neonatal pneumonia, a MAPK1 knockdown cell model was established. CCK-8 assay results indicated that both MAPK1 knockdown alone and the combination of MAPK1 knockdown with naringenin (50 μM) treatment significantly increased cell viability. Notably, cell viability in the combination treatment group was slightly lower than that in the MAPK1 knockdown alone group (Figure 8A). RT-qPCR analysis demonstrated that, compared with the si-NC groups, MAPK1 knockdown significantly decreased the mRNA expression of pro-inflammatory cytokines (*p* < 0.0001) (Figure 8B). Western blotting analysis further confirmed these observations, showing that MAPK1 knockdown markedly downregulated the protein levels of the corresponding inflammatory factors (*p* < 0.0001) (Figure 8C). ELISA results revealed that MAPK1 knockdown, naringenin treatment alone, and their combined intervention all significantly reduced the levels of IL-6, IL-17, and TNF-α compared with the model group (*p* < 0.0001). Furthermore, naringenin treatment alone yielded slightly lower cytokine levels than MAPK1 knockdown or the combined treatment, implying that the anti-inflammatory effect of naringenin may be partially independent of MAPK1 (Figure 8D). Taken together, these findings demonstrate that MAPK1 knockdown suppresses inflammatory cytokine secretion and improves cell viability in neonatal pneumonia-related cells.

## 4 Discussion

To address the limitations of existing studies on TCM for NP, this study adopted an integrated strategy combining meta-analysis, network pharmacology, molecular docking, and *in vitro* validation. We systematically explored the potential active components, core targets, and underlying molecular mechanisms of Chinese patent medicines in treating NP, with a focus on the key component naringenin and the central target MAPK1. The core active components and their regulatory pathways were identified, providing reliable support for the clinical application of TCM in NP.

This meta-analysis based on multiple clinical studies investigating NP interventions systematically identified four medicinal herbs—Zisu, Chaihu, Bohe, and Jingjie—as promising therapeutic candidates. Evidence has validated the therapeutic potential of these herbs and their bioactive constituents against pneumonia. According to TCM theory, Zisu exerts effects of relieving exterior symptoms, dispelling cold, and harmonizing the middle energizer, and its main active components, such as perillaldehyde and rosmarinic acid, have been reported to possess anti-inflammatory, antibacterial, and antioxidant activities that could effectively alleviate airway inflammation (42–44). Chaihu, is an important TCM herb for relieving stagnation of liver qi and reducing fever, whose core active components are saikosaponins; it has functions of regulating immune response, inhibiting inflammatory factors, and protecting lung tissue (45–48). Additionally, as a classic herbal plant, Bohe has the effects of dispelling wind-heat, clearing the head and eyes, and relieving sore throat, and its main active components such as menthol and menthone have antiviral, antibacterial, and anti-inflammatory activities (49–51), while Jingjie exerts effects of relieving exterior symptoms, dispersing wind, and stopping bleeding; its active components have anti-inflammatory and antibacterial effects, which help alleviate inflammation caused by lung infection (52, 53). These research theories not only confirm the potential value of these Chinese herbal medicines in the treatment of NP but also provide a reasonable basis for taking them as research objects to further characterize their active components, core therapeutic targets, and molecular mechanisms.

Concurrently, 36 active components and 1,013 corresponding drug targets were screened from these four herbs using the TCMSP database; combined with 3,692 NP-related targets collected from GeneCards and OMIM databases, 163 common targets were identified through intersection analysis. Through STRING database upload, topological analysis of these common targets via cytoNCA plugin of Cytoscape identified six hub genes closely associated with NP pathogenesis (54, 55). Furthermore, module analysis using the MCODE plugin identified two key functional subnetworks. Combined with the results of GO and KEGG enrichment analyses and the therapeutic direction confirmed by meta-analysis, MAPK1 was selected as the primary hub target for subsequent experimental validation, given its high topological significance and close association with the inflammatory response in NP. Molecular docking represents a powerful approach for predicting the binding affinity between small-molecule compounds and target proteins, providing a structural basis for investigating direct interaction mechanisms. In the present study, molecular docking was performed between MAPK1 and four representative active components, among which naringenin exhibited the strongest binding stability with MAPK1, suggesting that naringenin may directly bind to MAPK1 and regulate its biological activity. Naringenin is a natural flavonoid compound widely present in various Chinese herbal medicines, with a stable chemical structure. It mainly exists in the form of naringin, and after entering the body, it could be converted into a biologically active free form through metabolism by intestinal flora or hepatic enzymatic hydrolysis, thereby exerting pharmacological effects (56, 57). Notably, the chemical composition of bohe, which was selected through the meta-analysis in this study, has been reported to contain the flavonoid compound naringenin (58–60). Naringenin possesses a variety of significant pharmacological activities; in addition to the classic antioxidant, anti-inflammatory, and antibacterial effects, it has also been confirmed to have biological properties such as regulating immune function and inhibiting the excessive activation of inflammatory signaling pathways (61–63). These properties are highly consistent with the pathogenesis of NP, such as imbalanced inflammatory responses and immune disorders, further supporting its good application potential in the treatment of NP.

As a core component of the MAPK/ERK signaling pathway, MAPK1 also functions as a pivotal regulator of inflammatory responses and intracellular homeostasis (64, 65). During the progression of NP, pathogenic infection such as bacterial LPS stimulation induces excessive activation of the MAPK1 pathway, which further triggers downstream signaling cascades to promote the release of pro-inflammatory cytokines including IL-6, TNF-α, and IL-1β, ultimately leading to pulmonary epithelial barrier disruption, interstitial edema, and tissue injury (66). Furthermore, the systemic inflammatory response driven by overactivated MAPK1 is closely associated with the development of severe NP complications such as bronchopulmonary dysplasia (67), highlighting the central role of MAPK1 in disease pathogenesis through its regulation of inflammatory factor release and promote inflammatory signaling. Accordingly, MAPK1 has been regarded as a promising therapeutic target for pneumonia and other inflammatory lung diseases (68). Our *in vitro* results further demonstrated that MAPK1 knockdown significantly reduced the mRNA and protein levels of IL-6, IL-17, and TNF-α in LPS-stimulated BEAS-2B cells, thereby effectively attenuating the inflammatory response and improving cell viability. These findings not only verify the pathogenic role of MAPK1 in NP but also suggest the feasibility of targeting MAPK1 for NP treatment. Meanwhile, our data also show that naringenin dose-dependently enhances cell viability and downregulates the expression of pro-inflammatory cytokines. Zhu et al. (69) found that naringenin could cross the blood-brain barrier and attenuate neuroinflammatory responses both *in vivo* and *in vitro* by inhibiting the MAPK signaling pathway. Zhao et al. (70) confirmed that naringenin inhibits the expression of pro-inflammatory mediators in RAW264.7 macrophages by targeting the MAPK pathway and reducing the phosphorylation level of p38, thereby exerting an anti-inflammatory effect. Consistent with these results, our *in vitro* experiments further verified that the regulatory effect of naringenin on cell viability and inflammatory response is closely associated with the MAPK signaling pathway; knockdown of MAPK inhibits the resistance effect of naringenin on inflammatory factors, indicating that the regulation of the MAPK signaling pathway may be the core molecular mechanism underlying its therapeutic effect. Notably, the inhibitory effect of naringenin alone on the release of pro-inflammatory cytokines is stronger than that of MAPK1 knockdown alone or the combined treatment of MAPK1 knockdown plus naringenin, suggesting that the anti-inflammatory activity of naringenin in NP may be partially independent of MAPK. Overall, these results not only provide experimental evidence for the therapeutic mechanisms predicted by our meta-analysis and network pharmacology but also further support the multi-target and multi-pathway regulatory characteristics of TCM.

Despite our integrated strategy systematically revealing the mechanisms of Chinese patent medicines in the treatment of NP, several limitations remain. The number of literatures included in the meta-analysis is relatively small, which may be affected by publication bias; all experiments were conducted only *in vitro*, lacking *in vivo* validation; the detailed mechanism by which naringenin regulates MAPK1 remains unclear; and the synergistic therapeutic effects among other multiple active components have not been explored. Future studies will further optimize the meta-analysis by expanding the scope of included studies and incorporating unpublished data; establish neonatal pneumonia animal models for *in vivo* validation, clarify the molecular mechanism of the luteolin/MAPK1 interaction, investigate the synergistic effects of multiple components, and expand database mining to identify more potential targets. Additionally, the combination of Chinese herbal components with antibiotics to improve therapeutic efficacy and reduce drug resistance, where possible, is worthy of further investigation.

## 5 Conclusion

In conclusion, this study systematically analyzed and validated the core active components and mechanisms of TCM in the treatment of NP through an integrated strategy. Specifically, the results confirmed the clinical applicability of the four core TCM herbs, verified the potential of naringenin as a candidate adjuvant therapeutic agent for NP, and identified MAPK1 as the core target through which naringenin exerts its therapeutic effects. Meanwhile, this study provides a practical methodological paradigm for exploring the multi-component and multi-target mechanisms of TCM in complex diseases, laying a theoretical foundation for the clinical translation of TCM in NP treatment and offering a new direction for the research on targeted therapy of NP.

## Data Availability

The original contributions presented in the study are included in the article/Supplementary Material, further inquiries can be directed to the corresponding author.
